# Donor Death Category Is an Effect Modifier Between Cold Ischemia Time and Post-transplant Graft Function in Deceased-Donor Kidney Transplant Recipients

**DOI:** 10.3389/fmed.2021.743085

**Published:** 2021-11-23

**Authors:** You Luo, Zhanwen Dong, Xiao Hu, Zuofu Tang, Jinhua Zhang, Weiming Deng, Xiangling Wei, Bin Miao, Feng Qin, Ning Na

**Affiliations:** ^1^Department of Kidney Transplantation, The Third Affiliated Hospital of Sun Yat-sen University, Guangzhou, China; ^2^Department of Neurosurgery, The Third Affiliated Hospital of Sun Yat-sen University, Guangzhou, China

**Keywords:** kidney transplantation, cold ischemia time, delayed graft function, DCD, DBD, effect modification

## Abstract

**Objectives:** We aimed to analyze the effect of cold ischemia time (CIT) on post-transplant graft function through mixed-effect model analysis to reduce the bias caused by paired mate kidneys.

**Methods:** We reviewed all kidney transplantation records from 2015 to 2019 at our center. After applying the exclusion criteria, 561 cases were included for analysis. All donor characteristics, preservation and matching information, and recipient characteristics were collected. Transplant outcomes included delayed graft function (DGF) and estimated glomerular filtration rate (eGFR). Generalized linear mixed models were applied for analysis. We also explored potential effect modifiers, namely, donor death category, expanded criteria donors, and donor death causes.

**Results:** Among the 561 cases, 79 DGF recipients developed DGF, and 15 recipients who died after surgery were excluded from the eGFR estimation. The median stable eGFR of the 546 recipients was 60.39 (47.63, 76.97) ml/min/1.73 m^2^. After adjusting for confounding covariates, CIT had a negative impact on DGF incidence [odds ratio = 1.149 (1.006, 1.313), *P* = 0.041]. In the evaluation of the impact on eGFR, the regression showed that CIT had no significant correlation with eGFR [β = −0.287 (−0.625, 0.051), *P* = 0.096]. When exploring potential effect modifiers, only the death category showed a significant interaction with CIT in the effect on eGFR (*P*_interaction_ = 0.027). In the donation after brain death (DBD) group, CIT had no significant effect on eGFR [β = 0.135 (−0.433, 0.702), *P* = 0.642]. In the donation after circulatory death/donation after brain death followed by circulatory death (DCD/DBCD) group, CIT had a significantly negative effect on eGFR [β= −0.700 (−1.196, −0.204), *P* = 0.006]. Compared to a CIT of 0–6 h, a CIT of 6–8 or 8–12 h did not decrease the post-transplant eGFR. CIT over 12 h (12–16 h or over 16 h) significantly decreased eGFR. With the increase in CIT, the regenerated eGFR worsened (*P*_trend_ = 0.011).

**Conclusion:** Considering the effect of paired mate kidneys, the risk of DGF increased with prolonged CIT. The donor death category was an effect modifier between CIT and eGFR. Prolonged CIT did not reduce the eGFR level in recipients from DBDs but significantly decreased the eGFR in recipients from DCDs/DBCDs. This result indicates the potential biological interaction between CIT and donor death category.

## Introduction

Kidney transplantation is an effective alternative to dialysis treatment for end-stage renal disease patients and has advantages over it. Currently, expanded criteria donors are gradually being accepted to expand the donor pool. Cold ischemia time (CIT) is one of the numerous factors affecting transplant outcomes. It is generally considered that acute kidney injury induced by ischemia is the cause of delayed graft function (DGF) (1, 2). Ischemia time naturally affects the degree of acute kidney injury, thus possibly affecting transplant outcomes. However, the effect of CIT on transplant outcomes is controversial in the literature. Foroutan et al. performed a systematic review of risk factors for 1-year graft survival and observed no or little effect of CIT on graft survival (3). The unit analyzed in their study was each increased hour of CIT. Large cohorts from the United Kingdom and the Scientific Registry of Transplant Recipients (SRTR) in the United States have shown that CIT did not impact graft survival (4–7). In other studies, prolonged CIT significantly reduced graft survival (8–13). The inconsistency of these results may result from how different populations are affected by local public policies, the sample size, the analytic methods and models, or other causes of bias.

Regarding the analytic methods, previous studies have neglected the pre-conditions of case independence in their multivariate regression adjustments. There exists a similitude effect of transplant outcomes in both recipients of mate kidneys. In other words, the graft recovery in both recipients of mate kidneys is not independent because they share mate kidneys from the same donor. There are several methods to address cluster data, namely, robust cluster standard error estimation, mixed effect models, generalized estimating equations, and multistate analysis (14). In this article, we aimed to analyze the effect of CIT on post-transplant graft function through mixed-effect model analysis to reduce the bias caused by paired mate kidneys.

## Methods

### Study Design and Patients

**Figure 1:**
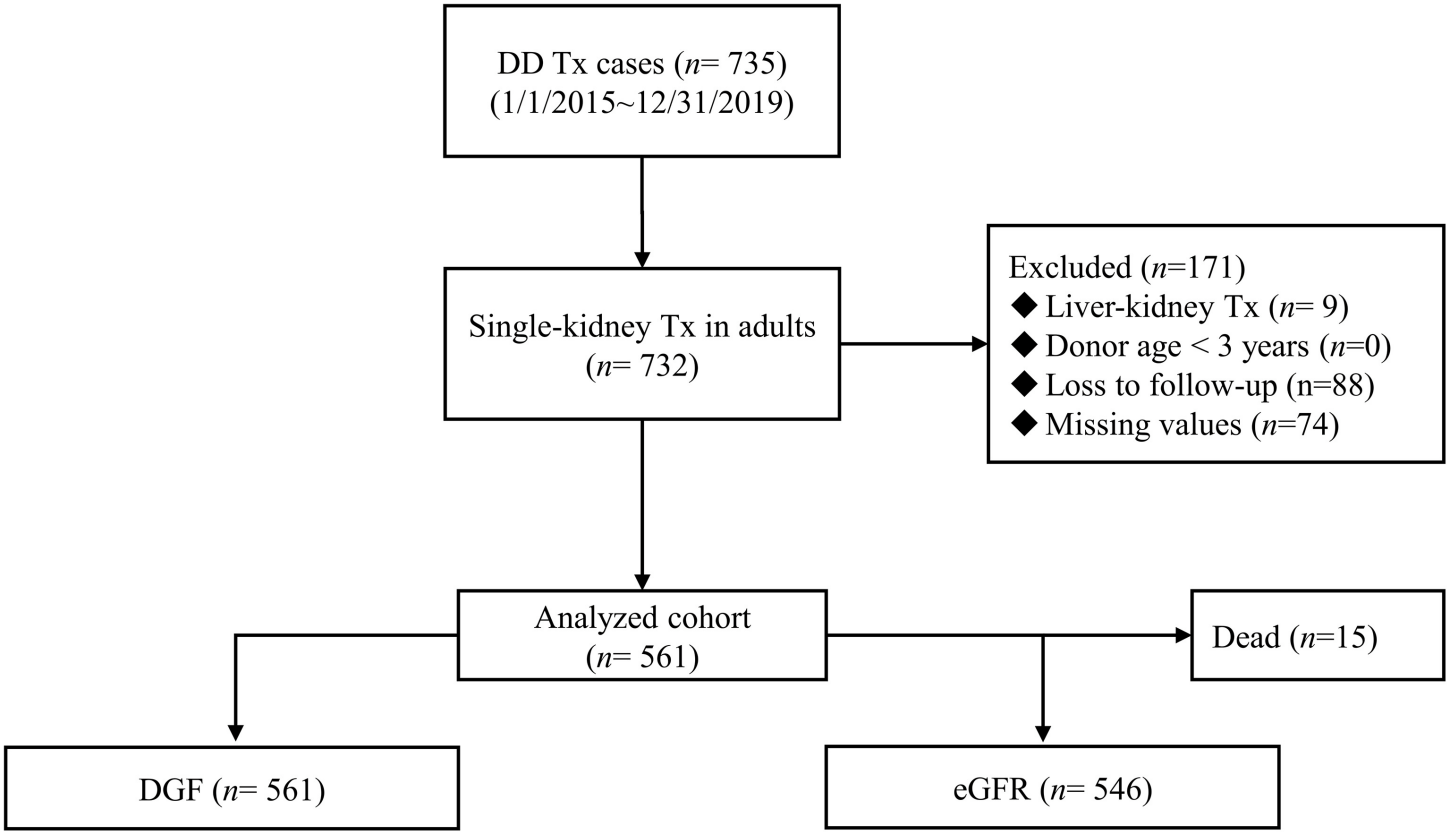
The recipient screening process for analysis. DD, deceased donor; Tx, transplantation.

The China Organ Transplant Response System (COTRS) is the sole legitimate official registry designated by the National Health Commission of China for solid organ donation, matching, and allocation. It includes a potential donor registry system maintained by the donation coordinators in each organ procurement organization (OPO), an allocation system maintained by the OPO (merged), and a waiting list maintained by each transplant registry center. We accessed and retrieved cases in our transplant center from January 1, 2015 to December 31, 2019. The inclusion criteria were single-kidney transplantation, donor age ≥ 3 years, and recipient age ≥ 18 years. Any recipient who was lost to follow-up was excluded. The detailed screen diagram is shown in Figure 1. All donors were deceased citizen donors, as this is the only legal avenue for non-relative solid organ transplantation procurement in the Chinese mainland as of January 1, 2015. All donors and procured organs were numbered, matched, and allocated at COTRS (https://www.cot.org.cn/). This study was approved by the research ethics committee at the Third Affiliated Hospital of Sun Yat-sen University [IRB Approval: (2020)02-243-01]. Donor profile access was permitted after ethical review. This research complied with the Declaration of Helsinki. The clinical and research activities reported here are consistent with the Principles of the Declaration of Istanbul as outlined in the “Declaration of Istanbul on Organ Trafficking and Transplant Tourism.”

### Data Sources and Variables

The primary outcomes of this research comprised DGF and stable graft function [estimated glomerular filtration rate (eGFR)] after transplantation. DGF was defined as the need for dialysis during the 1st week after transplantation (15). Graft recovery function (i.e., stable eGFR) was manually evaluated at each follow-up visit. It was estimated by the range of creatinine values within the 1st year. We used the median creatinine value as the index of stable function and calculated the eGFR by the Chronic Kidney Disease Epidemiology Collaboration formula (16). All data were from COTRS (including its waitlist and allocation system), medical records, tests, and examinations from the donation hospitals and transplant hospitals. These data were legally protected and inspected for their authenticity. The authors were authorized to retrieve the relevant data from these platforms.

Common variables of donors included age, sex, blood type, height, weight, terminal serum creatinine, and cause of death. Expanded criteria donors were defined using Rao's definition (17). The donor death category was divided into three classifications: donation after brain death (DBD), donation after circulatory death (DCD), and donation after brain death followed by circulatory death (DBCD) (18). Because the sample size of DBCD was small, we merged DCD and DBCD into the single group DCD/DBCD. CIT was estimated as the interval from donor death to reperfusion of the anastomotic kidney, ignoring the short duration (several minutes) between death and initial cold perfusion in the donor. All donated kidneys were placed in static cold storage and transported. Common variables of recipients included recipient age, sex, height, weight, diabetes, dialysis mortality, dialysis vintage, and peak panel reactive antibody.

### Statistical Analysis

Categorical variables are described as percentages and were compared between groups by the Fisher's exact-test or the chi-squared-test. Continuous variables are expressed as the mean and standard deviation (for normal distributions) or median and interquartile range (for skewed distributions) and were compared by the Wilcoxon test. To demonstrate the impact of CIT on transplant outcomes, considering the intercorrelation within mate kidneys from the same donor, we used mixed-effect models for analysis. The random variable was donor ID code. In addition, we used the *P*-values of the interaction and the effect size in each subgroup to explore the effect-modifying factors. Potential factors included donor death category, expanded criteria donors, and donor death causes. A mixed logit model was used for binary outcomes, and mixed linear regression was applied for continuous outcomes. The standard error of each coefficient was calculated *via* robust estimation. All statistical analyses were performed using Stata 16.1 IC (StataCorp, College Station, TX, USA) and R version 4.1.0 (R Foundation for Statistical Computing, Vienna, Austria). A two-sided *P*-value < 0.05 was considered statistically significant.

## Results

### Baseline Demographics

From 2015 to 2019, 735 recipients had deceased-donor kidney transplantation records. After excluding child recipients (*n* = 3), liver-kidney transplantations (*n* = 9), recipients lost to follow-up (*n* = 88), and donors with key values not reported in COTRS (missing values due to no access to other OPOs, *n* = 74). A total of 561 kidney transplant cases were remaining for analysis. Among them, 15 recipients died in the post-operative period, which was attributed to transplant failure (Figure 1). The baseline characteristics of the analyzed cohort are listed in Table 1. There were 79 DGF recipients and 15 recipients who died after surgery. The median stable eGFR of the 546 recipients was 60.39 (47.63, 76.97) ml/min/1.73 m^2^. The stable post-transplant eGFR was much lower in DGF recipients (*P* < 0.001) than in non-DGF recipients. In addition, DGF recipients had higher body weight, longer dialysis duration, more hemodialysis modalities, longer CIT, higher donor body height and weight, higher terminal serum creatinine level, higher cerebrovascular death rate, and higher expanded criteria donors (ECD) rate.

**Table 1:** Demographic summary of the analyzed cohort.

**Risk factors**	**Non-DGF (***N*** = 482)**	**DGF (***N*** = 79)**	***P*** **-values**
eGFR (*n* = 546)	62.26 (49.43, 78.44)	46.33 (36.95, 62.41)	**<0.001**
Cold ischemia time (CIT, hours)	8.2 (6.8, 10.3)	9 (7, 11.8)	**0.021**
CIT category			0.120
0–6 h	69 (14.3%)	8 (10.1%)	
6–8 h	160 (33.2%)	23 (29.1%)	
8–12 h	173 (35.9%)	29 (36.7%)	
12–16 h	57 (11.8%)	9 (11.4%)	
>16 h	23 (4.8%)	10 (12.7%)	
Sex			0.081
Female	145 (30.1%)	16 (20.3%)	
Male	337 (69.9%)	63 (79.7%)	
Age (years)	42 (33, 51)	43 (34, 47)	0.900
Height (cm)	168 (164, 172)	169 (166, 172)	0.085
Weight (kg)	60 (53.5, 69)	65 (56, 73.5)	**0.002**
BMI	21.3 (19.3, 23.8)	22.9 (19.6, 25.8)	**0.008**
Diabetes			0.594
No	419 (86.9%)	67 (84.8%)	
Yes	63 (13.1%)	12 (15.2%)	
Dialysis modality			**0.003**
Preemptive transplant	54 (11.2%)	3 (3.8%)	
Hemodialysis	327 (67.8%)	68 (86.1%)	
Peritoneal dialysis	101 (21.0%)	8 (10.1%)	
Dialysis duration			**0.005**
0–6 months	184 (38.2%)	16 (20.3%)	
7–12 months	115 (23.9%)	22 (27.8%)	
>12 months	183 (38.0%)	41 (51.9%)	
PRA			0.567
Negative	426 (88.4%)	72 (91.1%)	
Positive	56 (11.6%)	7 (8.9%)	
Donor sex			0.086
Female	96 (19.9%)	9 (11.4%)	
Male	386 (80.1%)	70 (88.6%)	
Donor age (years)	44 (33, 52)	46 (37, 52)	0.072
Donor height (cm)	168 (162, 170)	170 (165, 173)	**0.004**
Donor weight (kg)	65 (56, 70)	68 (60, 75)	**0.002**
Donor BMI	22.6 (20.8, 24.5)	23.9 (21.3, 26.1)	**0.020**
Terminal serum creatinine (μmol/l)	107 (72, 177)	206 (113, 371)	**<0.001**
Donor hypertension			0.084
No	400 (83.0%)	59 (74.7%)	
Yes	82 (17.0%)	20 (25.3%)	
Death category			0.288
DBD	147 (30.5%)	19 (24.1%)	
DCD/DBCD	335 (69.5%)	60 (75.9%)	
Cause of death			**0.001**
Trauma/others	279 (57.9%)	30 (38.0%)	
Cerebrovascular	203 (42.1%)	49 (62.0%)	
Donor type			**0.036**
SCD	407 (84.4%)	59 (74.7%)	
ECD	75 (15.6%)	20 (25.3%)	

*Differences in continuous variables between groups were tested by the Wilcoxon-test. Fisher's exact-test was applied for categorical variables*.

*BMI, body mass index; DBCD, donation after brain death followed by circulatory death; DBD, donation after brain death; DGF, delayed graft function; eGFR, estimated glomerular filtration rate; PRA, panel reactive antibody. SCD, standard criteria donors; ECD, expanded criteria donors. Bold values indicate the statistical significance*.

### Cold Ischemia Time and Graft Function Recovery Outcomes

The mean CIT was 9.4 ± 4.1 h. Recipients with DGF had longer CIT (*P* = 0.021). After adjusting for confounding covariates, CIT had a negative impact on DGF incidence [odds ratio = 1.149 (1.006, 1.313), *P* = 0.041]. In the entire analyzed cohort of 546 cases, simple correlation analysis showed that CIT had no significant correlation with post-transplant eGFR (*P* = 0.410). Multivariate adjustment regression showed that CIT had no significant correlation with eGFR (Table 2). In addition, we divided CIT into several categories and performed multivariate regression analysis. The regression results are listed in Table 2. No significant effect was observed in any subgroup for either DGF or eGFR outcomes. The effect size trend of CIT on DGF was statistically significant (*P* = 0.044). As CIT increased, the effect size of DGF risk increased, although no significance was observed in any group compared to the CIT of 0–6 h.

**Table 2:** The impact of CIT on DGF and eGFR (based on mixed logit and linear model).

**Outcome**	**Risk factor**	**CIT category**	**β coefficient (95% CI)**	***P*** **-value**
DGF	CIT (hours)	Continuous	0.139 (0.006, 0.272)	**0.041**
	CIT (ref: 0–6 h)	6–8 h	−0.218 (−1.736, 1.300)	0.778
		8–12 h	0.005 (−1.440, 1.449)	0.995
		12–16 h	0.774 (−1.207, 2.756)	0.444
		>16 h	2.141 (−0.306, 4.588)	0.086
		*P* trend		**0.044**
eGFR	CIT (hours)	Continuous	−0.287 (−0.625, 0.051)	0.096
	CIT (ref: 0–6 h)	6–8 h	−0.171 (−4.725, 4.383)	0.941
		8–12 h	−1.265 (−5.813, 3.284)	0.586
		12–16 h	−2.251 (−8.029, 3.527)	0.445
		>16 h	−4.709 (−11.532, 2.114)	0.176
		*P* trend		0.136

*Adjusted confounding factors included recipients' sex, age, height, weight, diabetes history, dialysis modality, dialysis duration, and panel reactive antibody; and donor sex, age, height, weight, death category, terminal creatinine level, hypertension history, and cause of death. The random effect variable was donor ID number*.

*CIT, cold ischemia time; DGF, delayed graft function; eGFR, estimated glomerular filtration rate. Bold values indicate the statistical significance*.

### Potential Effect-Modifying Factors

**Figure 2:**
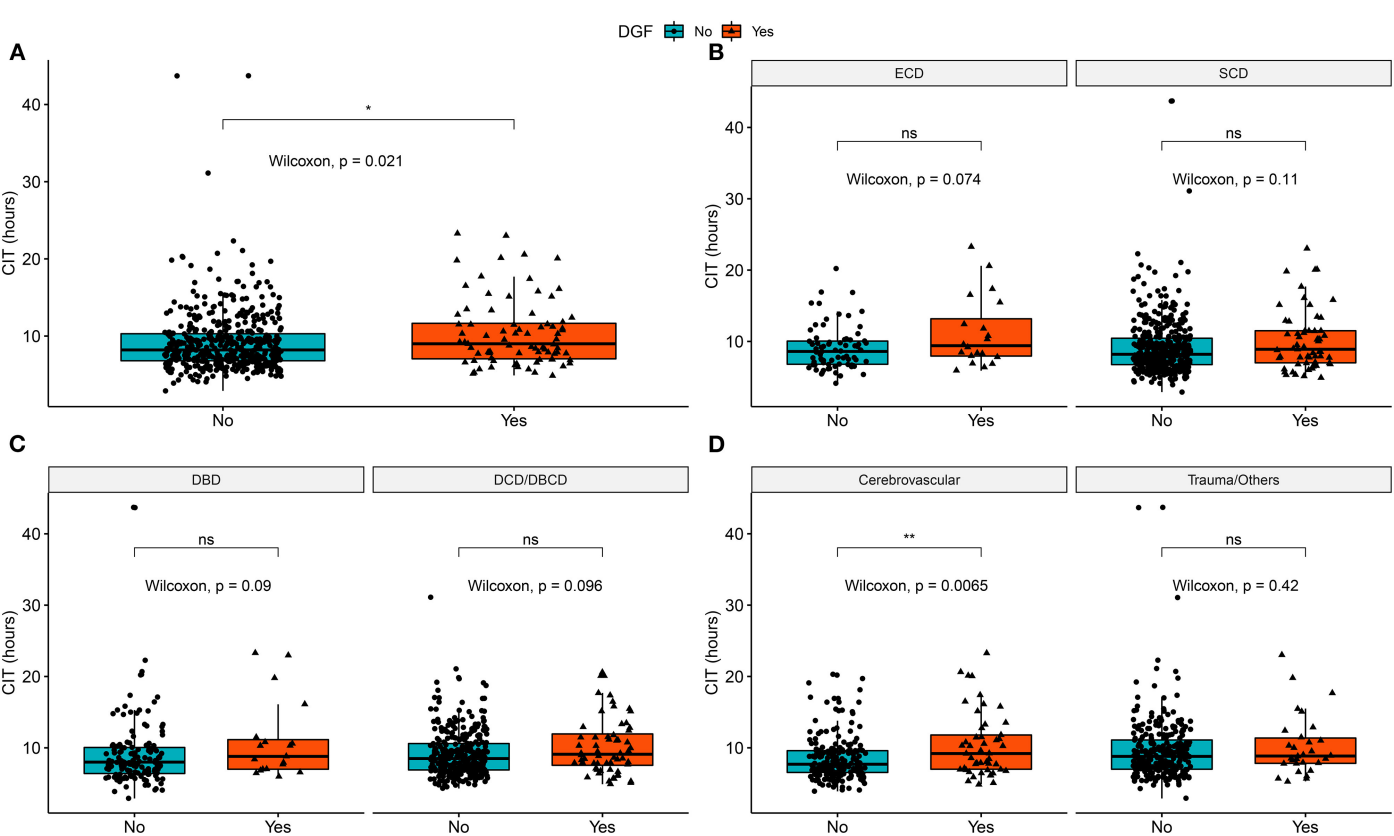
Distribution of cold ischemia hours in DGF/non-DGF groups in different subgroups and the entire cohort. **(A)** Cold ischemia time in the DGF and non-DGF groups (*P* = 0.021). **(B)** The difference in cold ischemia time between the DGF groups in SCD (*P* = 0.114) and ECD (*P* = 0.074). **(C)** The difference in cold ischemia time between DGF groups in DBD donors (*P* = 0.089) and DCD donors (*P* = 0.095). **(D)** The difference in cold ischemia time between DGF groups in donors who had cerebrovascular death (*P* = 0.007) and trauma/other cause of death (*P* = 0.423). DBD, donation after brain death; DCD, donation after circulatory death; DBCD, donation after brain death followed by circulatory death; SCD, standard criteria donors; ECD, expanded criteria donors; DGF, delayed graft function. *p < 0.05, **p < 0.01; ns, no significance.

We explored the correlation of CIT with DGF and eGFR in the entire cohort and each subgroup of potential effect-modifying factors. Figure 2 shows that in both donor type and death category stratifications, CIT was not significantly different between the DGF and non-DGF groups. In the cause of death subgroups (Figure 2D), recipients with DGF had longer CITs when the donor had cerebrovascular death. However, no differences were observed in the recipients from donors who had trauma/other causes of death.

**Figure 3:**
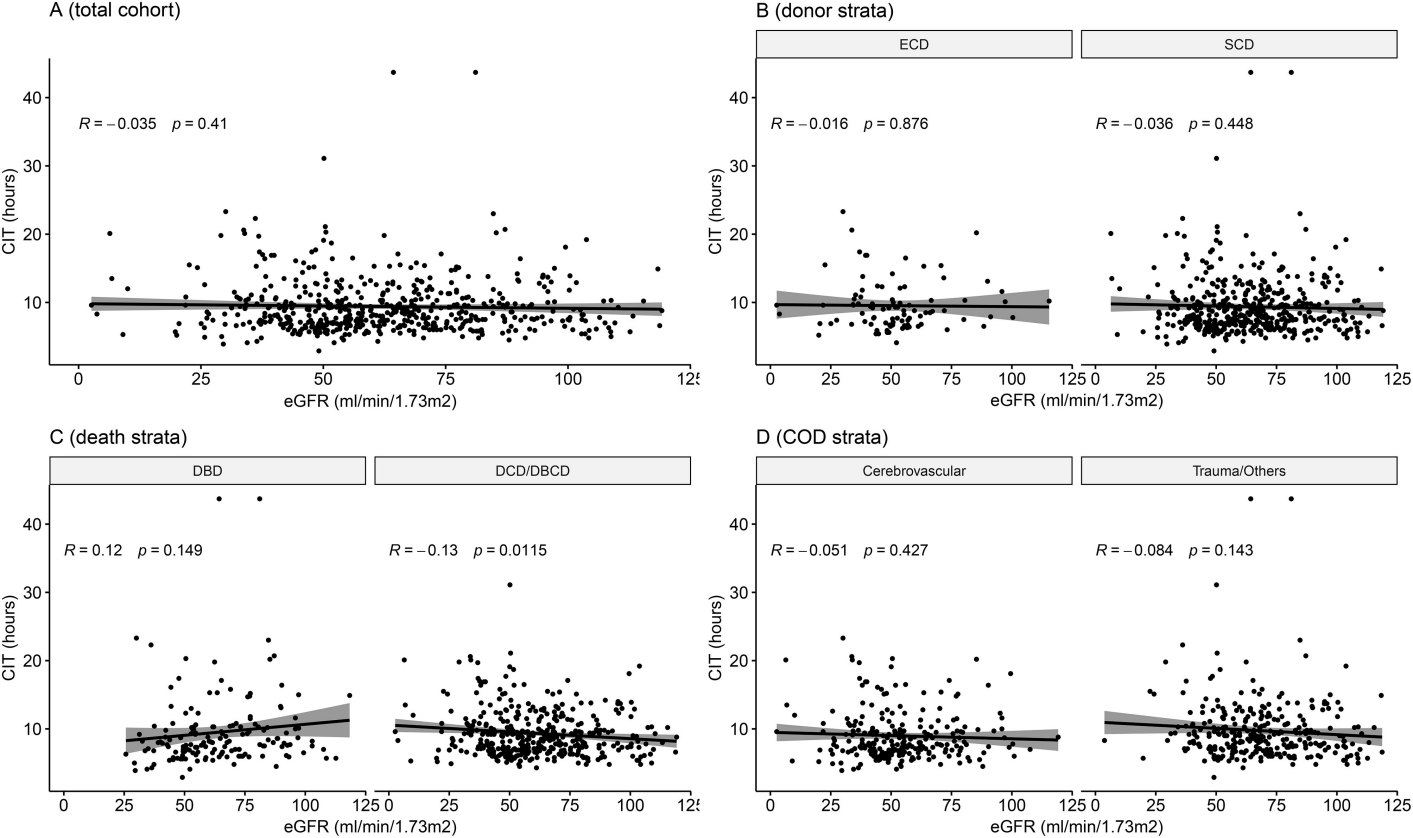
Correlation between cold ischemia time and eGFR in the entire cohort and in subgroups. **(A)** Entire cohort of 546 cases (*P* = 0.410). **(B)** Stratified by donor type (*P* = 0.876 in ECDs and *P* = 0.448 in SCDs). **(C)** Stratified by death category (*P* = 0.149 in DBDs and *P* = 0.012 in DCD/DBCDs). **(D)** Stratified by cause of death (*P* = 0.427 in the cardiovascular disease group and *P* = 0.143 in the trauma/other group). DBCD, donation after brain death followed by circulatory death; DBD, donation after brain death; DCD, donation after circulatory death; DBCD, donation after brain death followed by circulatory death; SCD, standard criteria donors; ECD, expanded criteria donors; eGFR, estimated glomerular filtration rate.

When exploring potential effect-modifying factors between CIT and eGFR, as shown in Figure 3, there was no correlation between CIT and eGFR in the global cohort, in each donor type or each cause of death strata. In the death category stratification, CIT was negatively correlated with eGFR in the DCD/DBCD group (*P* = 0.012), but there was no significant correlation in the DBD group (*P* = 0.149).

To quantitatively explore the existence of potential effect-modifying factors, we incorporated interaction terms in the regression model to test the interactions of CIT with donor type, death category, and cause of death. An interaction term with a *P*-value < 0.05 was considered to indicate an effect modification. Each regression result is listed in Table 3. Neither donor type nor the cause of death interacted with CIT. Only the death category showed a significant interaction with CIT on the eGFR outcome (*P*_interaction_= 0.027).

**Table 3:** Interactions between cold ischemia time (CIT) and potential effect-modifying factors.

**Risk factor**	**Outcomes**	**Donor type**	**Death category**	**Cause of death**
CIT (hours)	DGF	0.883	0.981	0.125
	eGFR	0.224	**0.027**	0.176

*All models were adjusted for the recipient's sex, age, height, weight, diabetes history, dialysis modality, dialysis duration, and panel reactive antibody; and donor sex, age, height, weight, death category, terminal creatinine level, hypertension history, and cause of death. The random effect variable was donor ID number*.

*DGF, delayed graft function; eGFR, estimated glomerular filtration rate. Bold values indicate the statistical significance*.

We further explored the effect size of CIT on eGFR in different death category strata (shown in Table 4). In the DBD group, CIT had no significant effect on eGFR (*P* = 0.642). In the DCD/DBCD group, CIT had a significantly negative effect on eGFR [β = −0.700 (−1.196, −0.204), *P* = 0.006]. Compared to a CIT of 0–6 h, a CIT of 6–8 or 8–12 h did not significantly increase the risk of eGFR recovery. CIT over 12 h (12–16 h or over 16 h) significantly decreased eGFR. With increasing CIT, the regenerated eGFR worsened. The trend test of effect size was significant (*P* = 0.011).

**Table 4:** The impact of CIT on eGFR stratified by donor death category.

**Stratification**	**Risk factor**	**CIT category**	**β coefficient (95% CI)**	***P*** **-value**
DBD	CIT (hours)	Continuous	0.135 (−0.433, 0.702)	0.642
	CIT (hours) (ref: 0–6 h)	6–8 h	1.685 (−4.625, 7.995)	0.601
		8–12 h	0.474 (−6.035, 6.982)	0.887
		12–16 h	7.214 (−2.505, 16.932)	0.146
		>16 h	0.619 (−11.087, 12.325)	0.917
		*P* trend		0.617
DCD/DBCD	CIT (hours)	Continuous	−0.700 (−1.196, −0.204)	**0.006**
	CIT (hours) (ref: 0–6 h)	6–8 h	−2.614 (−8.767, 3.538)	0.405
		8–12 h	−3.757 (−9.799, 2.286)	0.223
		12–16 h	−7.133 (−14.018, −0.247)	**0.042**
		>16 h	−9.253 (−17.775, −0.694)	**0.034**
		*P* trend		**0.011**

*Models were adjusted for recipients' sex, age, height, weight, diabetes history, dialysis modality, dialysis duration, and panel reactive antibody; and donor sex, age, height, weight, terminal creatinine level, hypertension history, and cause of death. The random effect variable was donor ID number*.

*DBCD, donation after brain death followed by circulatory death; DBD, donation after brain death; DCD, donation after circulatory death; eGFR, estimated glomerular filtration rate. Bold values indicate the statistical significance*.

## Discussion

In this study, we considered the effect of mate kidneys and found that prolongation of CIT increased DGF risk. In general, CIT did not correlate with the regenerated eGFR. However, we found that donor death category was an effect-modifying factor between CIT and eGFR. In DBD recipients, prolonged CIT did not reduce the eGFR level. In DCD/DBCD recipients, each additional hour of CIT significantly decreased eGFR by an average of 0.7 ml/min/1.73 m^2^.

It is common sense that CIT increases the risk of DGF, and our results bore this out. Our results did not reveal any interaction between CIT and donor type, death category, or cause of death when there was DGF. The eGFR decrease along with the CIT increase was modified by death category. In a previous publication, Wong et al. (19) proposed a significant interaction between donor death category and total ischemia time in the effect on death-censored graft loss (DCGL). There was a significantly increased risk of DCGL along with increased ischemia time in recipients of DCD kidneys [hazard ratio (HR), 1.08; 95% CI, 1.01–1.17; *P* = 0.03] but not in recipients of DBD kidneys (HR, 0.99; 95% CI, 0.98–1.01; *P* = 0.83). They also found that donor age was an effect modifier between ischemia time and eGFR at 12 months. The eGFR at 12 months decreased significantly with each ischemia hour in recipients of older donors but not in recipients of young donors (age <55 years). Our results agree with this conclusion when we treated donor age as a bivariate category with a cutoff of 55 years [β= −1.190 (−2.070, −0.310) ml/min/1.73 m^2^, *P* = 0.008 in recipients of donor age over 55 years and β = −0.204 (−0.570, 0.162) ml/min/1.73 m^2^ in recipients of donor age <55 years, *P* = 0.274; *P*_interaction_ = 0.014]. However, when we treated donor age as the original continuous variable, we did not find a significant interaction between CIT and donor age (*P*_interaction_ = 0.436). In addition, they did not explore whether the death category was an effect modifier. In another study, Summers et al. (20) also showed greater graft loss risk from cold ischemia prolongation in kidneys from DCD donors than in kidneys from DBD donors, especially when the CIT was over 24 h (*P*_interaction_ = 0.004); their study did not explore the effect modification by death category on eGFR recovery. Based on the previous consensus that eGFR recovery is a determining factor highly correlated with graft survival (21–25), the factors affecting graft survival very likely have the same impact trends on eGFR recovery. On this point, our results coincide with these other publications.

In a regression model, the presence of effect modification does not always imply biological interaction. However, a product term in a linear statistical model for a causal dependency can only arise from the presence of biological interaction in the dependent-action sense (26). Our study outcome was eGFR as a continuous variable, and the analytic model was a linear statistical model. Thus, we speculate that there was a potential biological interaction between the death category and CIT in the effect on eGFR recovery. The underlying mechanisms by which CIT influences transplant outcomes remain unclear. There exists a certain time-dependent molecular detriment mechanism since the effect of different CIT durations can be significantly different. Le Pape et al. (27) demonstrated that the unfolded protein response plays a critical role in elaborating the relationship between CIT and transplant outcomes. During the first 1–8 h, the eIF2a-ATF4 pathway is inhibited, while ATF6 is activated at 12–24 h and is associated with cell death. The IRE1a-XBP1 pathway is activated in reperfusion only if CIT surpasses 8 h, and IRE1a RNase activation is not detected before reperfusion but rather is only observable when preceded by at least 12 h of ischemia. These results coincide with our timeline trend outcomes in the subgroup analysis. Our results showed that in DCD recipients, CIT over 12 h (12–16 and >16) significantly decreased the eGFR compared to 0–6 h, while 6–12 h (6–8 and 8–12 h) did not. Our study showed that CIT was more detrimental to kidneys from DCD than to kidneys from DBD. How does the CIT interact with DCD, and does DCD or DBD trigger exacerbation or alleviation of subsequent detriment? The mechanism is still unclear. Saat et al. (28) compared kidney inflammatory, cytoprotective, and injury gene expression profiles from DCD and DBD in rat models. The results showed massive upregulation of proinflammatory genes, namely, IL-1β, IL-6, TNF-α, MCP-1, P-selectin, and E-selectin, in DBD kidneys compared with DCD kidneys immediately after kidney retrieval. During 18 h of cold ischemia, the expression levels of these genes did not significantly change. Interestingly, the expression of the cytoprotective gene heme oxygenase-1 (HO-1), whose upregulation is an adaptive response to oxidative stress, was significantly higher in DBD kidneys than in DCD kidneys during cold storage. A previous study demonstrated that HO-1 induction in brain-dead donors could improve allograft survival (29). In addition, the major difference between DCD and DBD kidneys is that DCD kidneys suffer worse warm ischemia injury before cold preservation. Warm ischemia time periods as short as a few minutes can lead to major metabolic changes. Baniene et al. (30) proved that warm ischemia leads to renal mitochondrial injury, which increases progressively with the increased duration of ischemia. As Saeb-Parsy et al. (31) summarized, mitochondria play central roles in ischemia-reperfusion injury and generate damaging reactive oxygen species. Mitochondrial injury promotes the release of damage-associated molecular patterns, which enhance inflammatory damage to the tissue. Thus, mitochondrial dysfunction caused by warm ischemia injury may induce susceptibility to cold ischemia and reperfusion injury. Mitochondrial transplantation significantly improves graft function and decreases graft tissue injury in a murine heart transplantation model (32). This evidence may explain why CIT had a more drastic impact on graft recovery in recipients from DCD.

Cold ischemia damage plays a critically important role in ischemia-reperfusion injury during kidney transplantation. Currently, there are some advances in reducing the detrimental effect of CIT on donors' kidneys. Hypothermic machine perfusion has been proven superior to static cold storage in terms of reducing DGF risk in deceased donor kidney transplantation (33). Modifying perfusion solution by adding agents against ATP depletion, Ca^2+^ overload, cell apoptosis, mitochondrial dysfunction, oxidative stress, inflammation, etc., is a practical direction to take (34). Gregorini et al. applied mesenchymal stromal cells (MSCs) and found that the addition of MSC/MSC-derived extracellular vesicles to Belzer solution during hypothermic machine perfusion protected the kidney from ischemic injury by preserving the enzymatic machinery essential for cell viability and protecting the kidney from reperfusion damage (35). To reduce the injury caused by hypoxia, oxygenated perfusion seems to be a reasonable alternative (36). However, current clinical trial results of oxygenated hypothermic machine perfusion are discouraging (36, 37). Normothermic machine perfusion (NMP) can mimic normal physiological perfusion of kidneys by oxygen carriers and can enable the reduction or avoidance of cold ischemia. Clinical trials assessing the effects of NMP on early and longer-term graft outcomes are needed (38). In addition, absorption of proinflammatory cytokines during cold perfusion breaks the cytokine cascade and amplification; absorption during perfusion reduces the inflammatory response and improves renal blood flow and graft viability in animal models (39, 40). It provides another means for reducing ischemia-reperfusion injury. By increasing the understanding of the potential mechanism of ischemia-reperfusion injury during kidney transplantation, therapeutic strategies based on different pathway targets are emerging. Our study revealed that DCD kidneys are more susceptible to cold ischemia damage than DBD kidneys. Although the underlying mechanism is unclear, understanding such a mechanism would provide new targets to reduce ischemia damage and improve DCD graft outcomes.

There are some limitations to this study. First, our sample was derived from our single transplantation center and had quite a few missing values or losses to follow-up. Second, the sample size was relatively small compared to the cohorts from transplant registry data. Finally, when we analyzed post-transplant eGFR, we excluded 15 recipients who died during the perioperative period after transplantation. These recipients would have had a lower eGFR if they had survived. This may have resulted in bias.

In conclusion, by considering the paired effect of mate kidneys, we showed that the risk of DGF increased with prolonged CIT. The donor death category was an effect modifier between CIT and eGFR. A longer CIT did not reduce the eGFR level in DBD recipients but significantly decreased the eGFR by an average of 0.7 ml/min/1.73 m^2^ with each prolonged CIT hour in DCD/DBCD recipients. This result indicates the potential biological interaction between CIT and donor death category.

## Data Availability Statement

The raw data supporting the conclusions of this article will be made available by the authors, without undue reservation.

## Ethics Statement

The studies involving human participants were reviewed and approved by Research Ethics Committee at the Third Affiliated Hospital of Sun Yat-sen University. Written informed consent for participation was not required for this study in accordance with the national legislation and the institutional requirements.

## Author Contributions

FQ and NN conceived and designed this study. YL, ZD, XH, ZT, JZ, XW, WD, and BM collected the data. YL and XH analyzed the data. YL and ZD wrote the manuscript. BM, NN, and FQ supervised the study and revised the manuscript. All authors contributed to the article and approved the submitted version.

## Funding

This work was supported by the National Natural Science Foundation of China (81970652), the Guangdong Basic and Applied Basic Research Foundation (2019A1515011219), the Bioengineering Research Center Training Project of the Third Affiliated Hospital of Sun Yat-sen University (SW201904), the Third Affiliated Hospital of Sun Yat-sen University Clinical Research Program (YHJH201906), and the Science and Technology Planning Project of Guangzhou (201803010016).

## Conflict of Interest

The authors declare that the research was conducted in the absence of any commercial or financial relationships that could be construed as a potential conflict of interest.

## Publisher's Note

All claims expressed in this article are solely those of the authors and do not necessarily represent those of their affiliated organizations, or those of the publisher, the editors and the reviewers. Any product that may be evaluated in this article, or claim that may be made by its manufacturer, is not guaranteed or endorsed by the publisher.
